# Assessment of HIF2α mutational pathogenicity using microscale thermophoresis

**DOI:** 10.1093/biomethods/bpag001

**Published:** 2026-01-13

**Authors:** Fraser G Ferens, Cassandra C Taber, Jeffrey J Eo, Michael Ohh

**Affiliations:** Department of Laboratory Medicine and Pathobiology, Faculty of Medicine, University of Toronto, 1 King’s College Circle, Toronto, ON M5S 1A8, Canada; Department of Biochemistry, University of Alberta, 474 Medical Sciences Building, Edmonton, AL T6G 2H7, Canada; Department of Laboratory Medicine and Pathobiology, Faculty of Medicine, University of Toronto, 1 King’s College Circle, Toronto, ON M5S 1A8, Canada; Department of Laboratory Medicine and Pathobiology, Faculty of Medicine, University of Toronto, 1 King’s College Circle, Toronto, ON M5S 1A8, Canada; Department of Laboratory Medicine and Pathobiology, Faculty of Medicine, University of Toronto, 1 King’s College Circle, Toronto, ON M5S 1A8, Canada; Department of Biochemistry, Faculty of Medicine, University of Toronto, 661 University Avenue, Toronto, ON M5G 1M1, Canada

**Keywords:** microscale thermophoresis, HIF, PHD, Pacak–Zhuang syndrome

## Abstract

Pacak–Zhuang syndrome is an emerging pseudohypoxic disorder that causes defined but varied manifestations of neuroendocrine tumours with or without polycythemia or exclusively polycythemia. This disease is caused by mutations in the *EPAS1* gene, which encodes for one of three hypoxia-inducible factor (HIF) α subunits, HIF2α. As new mutations in this gene are observed in individuals exhibiting the manifestations of Pacak–Zhuang syndrome, there is a need to distinguish bona-fide disease causing mutations from benign mutations, which could have a valuable impact on the direction of patient care. We recently showed that reductions in the affinity of prolyl-hydroxylase 2 (PHD2) for HIF2α due to mutations are at the root of the mechanism underlying Pacak–Zhuang syndrome. The determination of affinity was accomplished using microscale thermophoresis (MST). Here, we describe a detailed protocol for the assessment of binding affinities between HIF2α peptides or the entire oxygen-dependent degradation domains of HIFα proteins and PHD2 using MST and propose that this method can be used to assess the potential pathogenicity of novel mutations in HIF2α.

## Introduction

**Figure 1:**
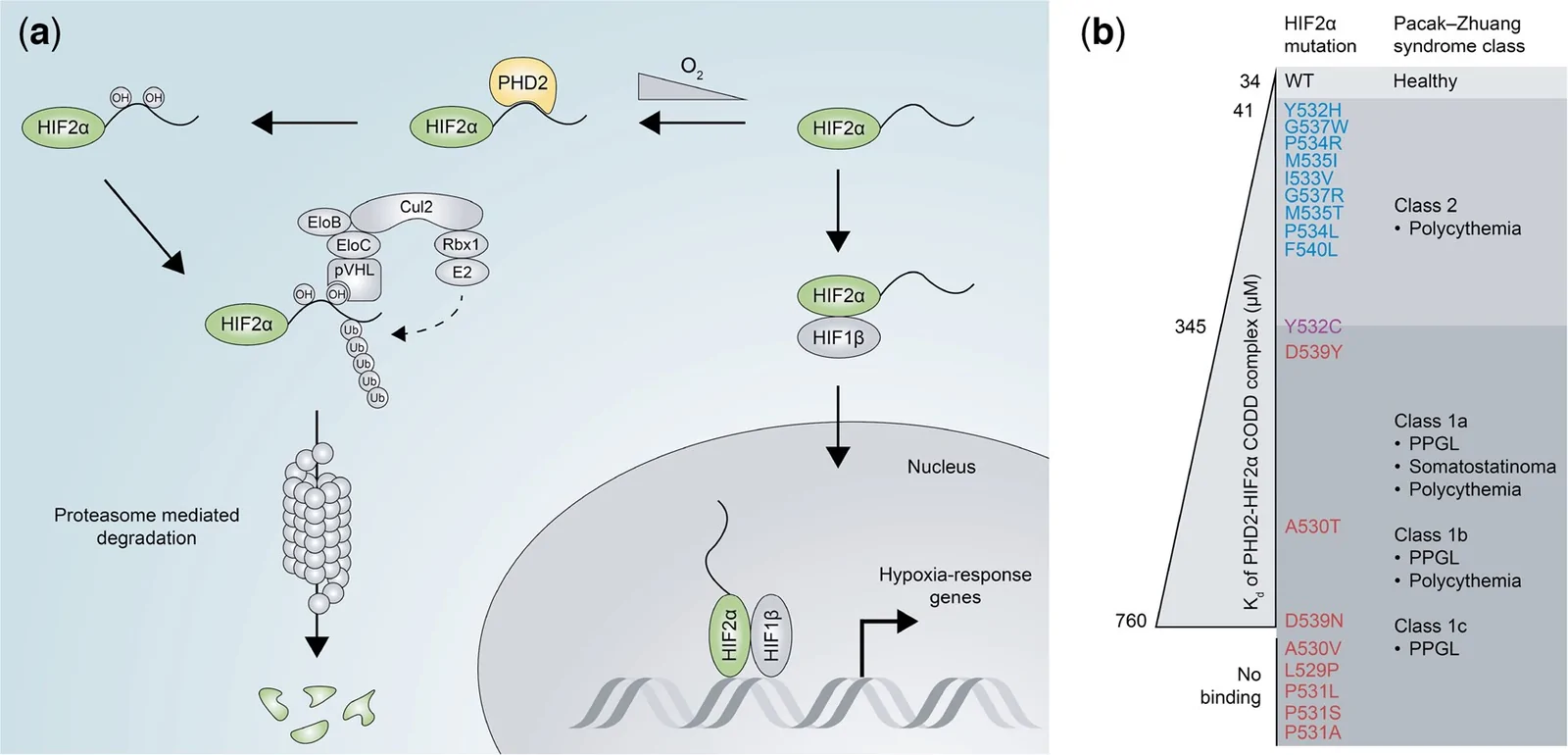
HIF2α in Pacak–Zhuang syndrome. (a) HIF2α regulation within the VHL-HIF pathway. In normoxia, PHD2 catalyses the hydroxylation of HIF2α at two proline sites, P405 and P531, using O_2_ as a co-substrate. Hydroxylated HIF2α is recognized by pVHL, a component of the pVHL-E3 ubiquitin ligase complex which contains Elongin B (EloB) and C (EloC), Cullin 2 (Cul2), RING-box protein 1 (Rbx1), and E2 ubiquitin ligase. This recognition leads to the ubiquitination (Ub) and subsequent proteasomal degradation of HIF2α. In hypoxia limited oxygen concentrations hinder PHD2 catalytic activity, allowing HIF2α to escape degradation and dimerize with HIF1β to form the HIF2αβ (HIF2) heterodimer. As a dimer, HIF2 translocates into the nucleus and activates the transcription of hypoxia-response genes. (b) The binding affinity of mutated HIF2α CODD to PHD2 is correlative with Pacak–Zhuang syndrome class. HIF2α mutations observed in Pacak–Zhuang syndrome patients were assessed for their binding affinity to PHD2 by MST. Class 2 patient mutations (blue) are characterized by slightly lower *K_d’_s* compared to WT whereas Class 1 patient mutations (red) exhibit severe binding defects. One mutation, Y532C (purple), has been observed in both Class 1 and Class 2 patients [3, 12–14]. *K_d_* values shown here were observed in the context of HIF2α CODD peptides.

Mutations in the components of the metazoan oxygen-sensing pathway can be causative of pseudohypoxic cancer syndromes in humans. These cancer syndromes, VHL disease, Pacak–Zhuang syndrome and prolyl-hydroxylase (PHD)-driven disease are caused by mutations in the genes *VHL* [1, 2], *EPAS1* [3, 4] and *EGLN1* [5, 6], respectively and exhibit similar, defined but varied phenotypic presentations. The central components of this pathway are hypoxia-inducible factors (HIFs), dimeric transcription factors comprised of an oxygen-labile α subunit (HIFα) and a constitutively expressed β subunit (HIFβ), which govern the transcriptional response to changes in intracellular O_2_ concentration [7]. *EPAS1* encodes for one of three human HIFα paralogs, HIF2α, and *VHL* and *EGLN1* encode for the HIFα negative regulators pVHL and HIF PHD2, respectively. The canonical metazoan oxygen-sensing pathway regulates the stability and therefore activity of HIFs via destruction of HIFα through proteasome-mediated degradation in the presence of O_2_ [8]. The oxygen-dependent degradation of HIFα proteins is mediated by PHD enzymes which hydroxylate HIFα proteins at conserved proline residues within the oxygen-dependent degradation domain (ODD) utilizing O_2_ as a substrate for the reaction [9, 10]. pVHL is the substrate-conferring component of an E3 ubiquitin ligase (pVHL-E3) that binds to hydroxylated HIFα proteins and results in their poly-ubiquitylation [11] (Fig. 1a). Aberrant increases in HIFα stability, particularly HIF2α, have been observed to be a consequence of *bona fide* disease-causing mutations in this pathway with mounting evidence supporting the notion that the varied phenotypes observed in each individual disease are correlated to differential stabilization of HIFα proteins [15, 16]. This concept has been complicated by a lack of accuracy in distinguishing the effect on HIFα stability by computational or cell-based assays. Recently, we examined the effect that mutations in the HIF2α C-terminal ODD site (CODD) had on the affinity of its negative regulator PHD2 for HIF2α [16]. We found that the disease-associated mutations tested-to-date caused a measurable reduction in the affinity of PHD2 for HIF2α and that the disease phenotype was correlated with the magnitude of the decrease in the affinity (Fig. 1b).

**Figure 2:**
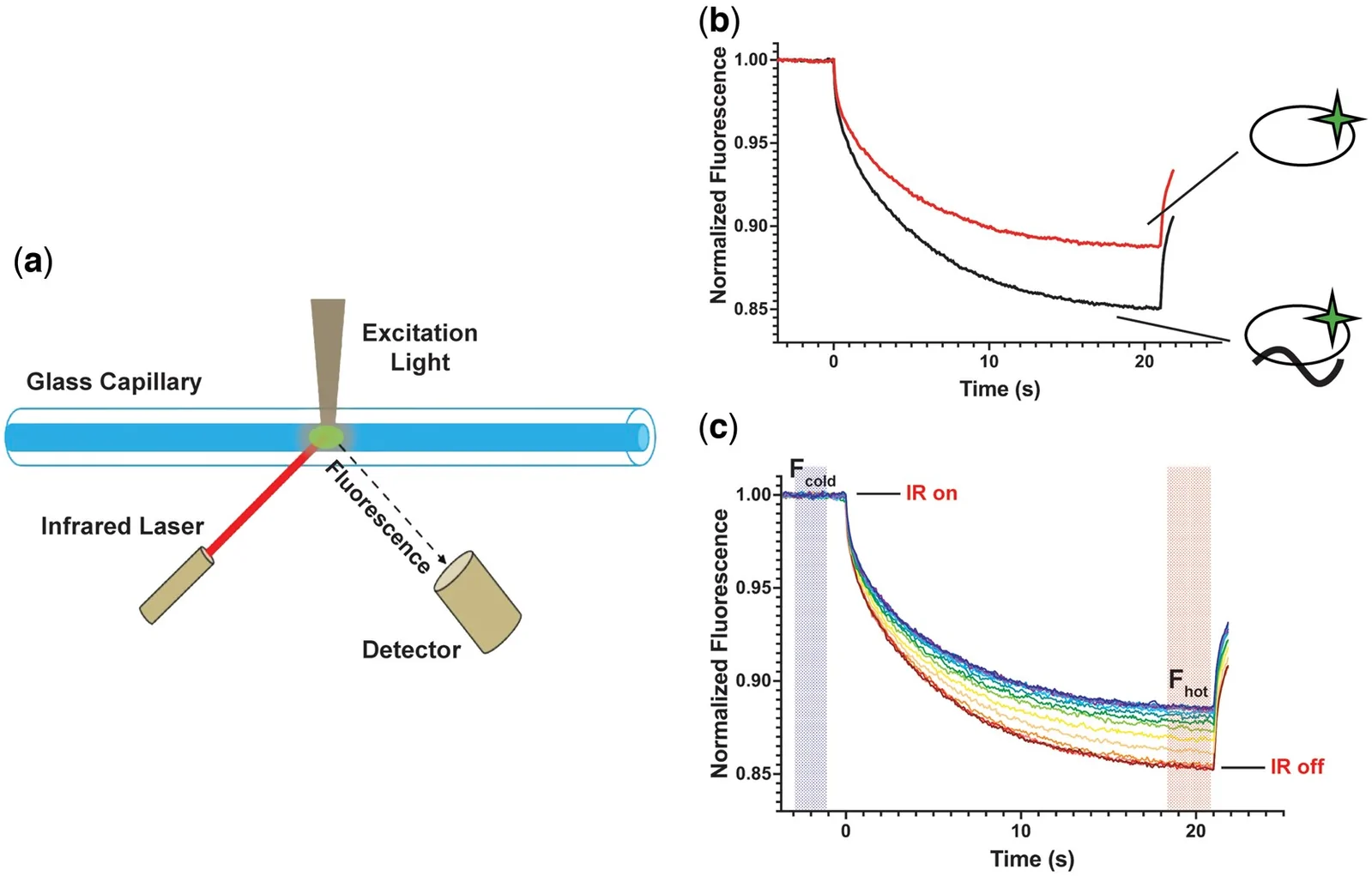
Principles of MST. (a) A glass capillary filled with a solution containing two molecules is heated in a localized area with an infrared (IR) laser. According to the phenomenon thermophoresis, molecules will move away from the heated area, creating a protein gradient. Molecular movement is tracked over time by a fluorescence detector aimed on the IR-heated area. (b) A binding test between PHD2–647 and a WT HIF2α CODD peptide was performed using MST. The red curve represents the time trace of PHD2–647 in the absence of WT HIF2α CODD while the black curve represents a time trace with PHD2–647 bound to WT HIF2α CODD. Images beside the graph illustrate PHD2–647 binding states. WT HIF2α CODD-bound PHD2–647 undergoes thermophoresis differently than unbound PHD2–647, creating different time traces that can be used to determine binding parameters. (c) WT HIF2α CODD peptide was titrated against a fixed concentration of PHD2–647 and samples from the titration series were analysed with MST, resulting in time traces that can be transformed into a binding curve by calculating the observed change in fluorescence (*F_n_*). *F_n_* is calculated by dividing the steady state fluorescence before the IR laser is turned off (*F*_hot_) by the initial fluorescence (*F*_cold_).

MST utilizes differences in the size and shape of macromolecules and macromolecular assemblies to determine binding affinity [17, 18]. This is accomplished via measurement of the movement of macromolecule mixtures in solution along a localized temperature gradient created by illuminating a small portion of the sample with an IR laser. Rapid formation of a localized temperature gradient induces localized motion of macromolecules away from the volume of elevated temperature until a new steady state is reached between the movement of molecules away from the heat source and the diffusion of molecules back into the volume of elevated temperature due to the decreased concentration of macromolecules in this volume relative to the surrounding solution [19] (Fig. 2a and b). The rate at which the system moves away from the initial fluorescence state (*F*_cold_) and approaches this steady-state (*F*_hot_) and the concentration of macromolecules within the volume of elevated temperature at steady state are relative to the physical properties of the molecules, that is, a complex of two proteins will have a different response to the creation of a localized temperature gradient than its individual components (Fig. 2b and c).

Thus, we can quantitatively determine the populations of individual proteins A and B along with their formed complex AB using a given set of solutions with known and varied concentrations of A and constant known concentration of B by measuring the thermophoretic response of protein B via fluorescent tag [20]. These quantifications allow for the determination of the binding affinity constant (*K*_d_) of the interaction between A and B. For the interactions discussed in this work, we will utilize the 1:1 *K*_d_ binding model:


$$F_{n}=\frac{F_{\mathrm{hot}}}{F_{\mathrm{cold}}},$$

(1) $$F_{n}({\left[A\right]}_{\mathrm{total}})=F_{B}+\;\frac{\left(F_{AB}-F_{B})({\left[A\right]}_{\mathrm{total}}+{\left[B\right]}_{\mathrm{total}}+K_{d}-\sqrt{{\left({\left[A\right]}_{\mathrm{total}}+{\left[B\right]}_{\mathrm{total}}+K_{d}\right)}^{2}-(4{\left[A\right]}_{\mathrm{total}}{\left[B\right]}_{\mathrm{total}})}\right)}{2{\left[B\right]}_{\mathrm{total}}},$$

Where *F_n_* is the observed change in fluorescence due to the activation of the IR laser relative to the initial fluorescence before activation (calculated according to Equation 1), *F_B_* is the relative change in fluorescence exhibited by the un-complexed labelled molecule, *F_AB_* is the relative change in fluorescence exhibited by the complex AB, [*A*]_total_ is the total concentration of the unlabelled molecule (i.e. [*A*]_total_ = [*A*] + [*AB*]), [*B*]_total_ is the total concentration the labelled molecule (i.e. [*B*]_total_ = [*B*] + [*AB*]) and *K_d_* is the dissociation constant. In this type of experiment, *F_B_*, *F_AB_* and *K_d_* are determined by fitting this model to measured *F_n_* values at a range of component A concentrations and constant concentration of fluorescently labelled protein B. A more thorough explanation of the derivation of this model as it pertains to MST can be reviewed in prior works [17, 18, 20].

### Advantages over other methods

There are many techniques available to researchers to measure interactions between molecules. The most popular of these are semiquantitative biochemical methods are tag-based pulldown or immunoprecipitation followed by immunoblotting or an enzyme-linked immunosorbent assay (ELISA). While these techniques have the benefits of low cost and effort, they are limited by specificity and quantification. In contrast, MST is highly sensitive and quantitative demonstrating an increased ability to distinguish between severe and minor HIF2α-PHD2 interaction defects compared to a previously devised in vitro hydroxylation assay [16, 21].

Additional biophysical techniques such as isothermal titration calorimetry (ITC), biolayer interferometry (BLI) or surface plasmon resonance (SPR) can also quantitively assess binding interactions, but they come with technical disadvantages such as high sample requirements (ITC) or tethered ligands (BLI and SPR). MST overcomes these complications with small sample requirements and a free solution environment that allows for random molecular diffusion. Additionally, MST experiments are simple to modify and relatively quick to perform, with little need for overnight incubations. For these reasons, MST is an ideal choice for measuring binding interactions between novel HIF2α mutants and PHD2 to efficiently predict pathogenicity.

### Experimental design

Typical MST experimental practice calls for nonfluorescent ligands to be titrated against a low, fixed concentration of fluorescently labelled ligands to measure binding events at equilibrium. Following this idea, the serial dilution described below should be carried out with nonfluorescent ligands (i.e. HIF2αCODD peptides and PHD2). The concentration of the fluorescent ligand should be lower than the expected *K_d_* to ensure 100% of the fluorescent ligand is bound even when varying amounts of its binding partner are present [22]. Minimum concentrations of the nonfluorescent ligand should be largely unassociated with the fluorescent protein and therefore produce MST responses indistinguishable from the unbound state of the fluorescent-protein. The maximum concentration of nonfluorescent ligand should be higher than the expected *K_d_* to reach binding saturation. Appropriate negative and positive controls should be derived from the same construct as experimental samples and should be prepared and tested under the same conditions. For our experiments, we utilized WT HIF2α constructs as positive controls and either hydroxylated HIF2α (HIF2α-OH) or hydroxy-proline mutant HIF2α constructs (P531A) as negative controls.

When attempting to determine the effect of mutation on the interaction between HIF2α and PHD2, the main consideration in peptide design will be the location of the mutation. Previously, we have examined the 20 amino acid (aa) sequence corresponding to the residues immediately surrounding HIF2αCODD hydroxy-proline site (ELDLETLAPYIPMDGEDFQL). This sequence contains the vast majority of known disease-causing mutations in HIF2α and serves as the primary interaction interface between HIF2α and PHD enzymes [15, 16]. Thus, any new mutations identified within this region should be assessed using modifications to this peptide sequence as there is already a large amount of available data that can aid in the interpretation of results. There have been reports of individuals experiencing the manifestations of Pacak–Zhuang syndrome with mutations in HIF2α outside this region yet still close in proximity to the 20 aa CODD site [23]. These types of mutations would warrant the inclusion of additional residues in the peptide sequence, though it should be noted that increasing the peptide length may alter the affinity of PHD enzymes for the peptide sequence [24]. Therefore, new wild-type and negative control peptides (such as P531S or P531-OH) should also be examined in parallel to determine relative differences in affinity, rather than comparing to shorter peptides. Even more distant mutations have been reported [25, 26] and may benefit from the examination of the interaction between PHD and the entire HIF2αODD domain.

The standard design for our peptides (ordered from GenScript) was to acetylate and amidate the N- and C-termini of the peptides respectively to avoid artefacts caused by inappropriate charges that would not be present in the full-length HIF2α protein. For the 20 aa HIF2αCODD region, the acetylated and amidated peptides were soluble directly in 50 mM Tris-HCl pH 8.0 buffer at a sufficient concentration for MST experiments (∼2 mM 2x Stock Solution), eliminating the need to introduce solvents such as DMSO to create our peptide stock solutions. Altering the length/sequence of the peptide may also alter the solubility of the peptide and therefore may necessitate re-optimization of various stages of the procedure.

MST experimental design should account for various technical considerations, including fluorescent dye selection, protein preparation, and buffer composition and inhibition of PHD2 enzymatic turnover which would result in the release of hydroxylated HIF2α from the enzyme. Fluorescent crosslinkers designed to interact with distinct functional groups are commonly used to label ligands. To fluorescently label PHD2, we used an N-hydroxysuccinimide (NHS) ester which crosslinks to primary amines on lysine residues, at the N-terminus or at guanidino groups on arginine residues [17]. Since proteins typically contain many primary amines, a dye may be cross-linked at different sites on each protein, but this potential variation in labelling site did not seem to impact our previous MST analyses of the WT PHD2 interaction with HIF2α peptides [16]. If a primary amine reactive cross linker dye is being used to examine the effects of HIF2α mutations on WT PHD2 interaction, we recommend that it be conjugated to WT PHD2 rather than the HIF2α peptide as the behaviour of labelled WT PHD2 has been previously characterized. The mutations of interest in the HIF2α sequence could potentially change how a cross linker dye labels the peptide; for example, a previous study suggested that crosslinking an NHS dye to PHD2 mutant P317R impacted HIF2α binding, falsely implying a severe binding defect, which was subsequently disproven through additional binding studies [27].

Careful preparation of an MST binding solutions is crucial to avoid issues such as noisy and deviant curves. Each time a protein component is thawed, it must be centrifuged to remove any particulate matter or aggregates that could interfere with MST signal, causing choppy curves or strong data variations between replicates. Additionally, a capillary scan should be performed to test solutions for surface adsorption. We have noticed that PHD2 and HIF2αODD-GFP adhere to MST capillary surfaces; our MST buffers have been supplemented with BSA or Tween20 to prevent sticking of the samples to capillary surfaces. If fluorophore contamination in BSA is a concern, a capillary scan comparing dilution buffer alone to dilution buffer with the labelled ligand can be performed to screen the level of background fluorescence. It should be noted that multiple types of glass capillaries are available, and we have used standard MST capillaries (NanoTemper) for all binding experiments. We recommend that PHD2 hydroxylation activity be inhibited using an α-ketoglutarate analogue which cannot be decarboxylated such as n-oxalylglycine but which still allows for the association of HIF2α with PHD2. Other methods of PHD2 inhibition such as the use of Mn^2+^ ions to compete with the bound Fe^2+^ ion in the PHD2 active site could also be explored as an alternative to stabilize the PHD2-HIF2α interaction [16]. Additional optimization suggestions and general MST practice can be found in detail in the previous work by Seidel *et al*. [17].

## Materials

### Plasmids

pET-46-His_6_-PHD2: pET vector encoding PHD2 (181–426) with a His_6_ tag [15].

pET21b-HIF2α-cp8GFP-35-His_6_: pET Vector encoding HIF2α-ODD fused to a circular permutant of super-folder eGFP followed by a 35 amino acid disordered tail and a His_6_ tag [28].

### General solutions and reagents

Competent *E. coli* BL21(DE3) cells

LB Broth Lennox (Bioshop, LBL405.500)

Ampicillin (Bioshop, AMP201.100)

Isopropyl β-D-1-thiogalactopyranoside (IPTG) (Bioshop, IPT002.25)

SigmaFast Protease Inhibitor (Sigma-Aldrich, S8830)

Tris Base (Bioshop, TRS001.5)

Sodium Chloride (NaCl) (Bioshop, SOD001.10)

Imidazole (Bioshop, IMD508.500)

Hydrochloric Acid (Caledon, 6025-1-29)

4-(2-Hydroxyethyl)piperazine-1-ethanesulfonic acid (HEPES) (Bioshop, HEP001.500)

His-Pur^TM^ Ni-NTA Resin (Thermo Scientific, 88222)

Thrombin (Sigma-Aldrich, T4648)

Dimethyl Sulfoxide (DMSO) (Fisher, D1361)

Alexa Fluor 647 NHS ester dye, 3 × 100 µg (Thermo Scientific, A37573)

Liquid nitrogen

2-mercaptoethanol (Bioshop, MER002.100)

Bovine serum albumin (BSA) (Bioshop, ALB001.250)

Tween20 (Bioshop, TWN510.500)

Iron (II) sulphate heptahydrate (FeSO_4_) (Sigma-Aldrich, 215422)

N-oxalylglycine (NOG) (Sigma-Aldrich, O9390)

### Laboratory equipment

−80°C Freezer

Bacterial Shaker

Avanti JXN-26 Centrifuge (Beckman Coulter)

Mechanical cell disruptor (we used an Emulsiflex C3 [Avestin] but a similar device or French Press should also be appropriate)

Nanodrop spectrophotometer (Thermo Scientific)

SnakeSkin^TM^ dialysis tubing 10 000 Molecular Weight Cut Off (Thermo Scientific, 68100)

Amicon^®^ Ultra Centrifugal Filter, 10 kDa Molecular Weight Cut Off (Sigma-Aldrich, UFC901008)

Superdex 75 10/300 GL Increase Column (Cytiva, 29148721)

AKTApurifier (Cytiva)

PD-10 desalting column containing Sephadex G-25 resin (Cytiva, 17085101)

Superdex 200 10/300 GL increase column (Cytiva, 28890944)

Pierce BCA protein assay kit (Thermo Scientific, 23227)

MST instrument (such as Monolith NT.115) (Nanotemper Technologies)

Standard MST capillaries (Nanotemper Technologies, MO-K022)

## Methods

### Preparation of protein samples

#### Purification of PHD2 catalytic domain

##### Buffers

Lysis Buffer (50 mM Tris-HCl, 500 mM NaCl, 5 mM Imidazole pH 7.9)

Wash Buffer (50 mM Tris-HCl, 500 mM NaCl, 30 mM Imidazole pH 7.9)

Ni-NTA Elution Buffer (50 mM Tris-HCl, 500 mM NaCl, 1 M Imidazole pH 7.9)

Dialysis Buffer (50 mM Tris-HCl, pH 7.5)

SEC Buffer (50 mM HEPES, 100 mM NaCl, pH 7.5)

**Figure 3:**
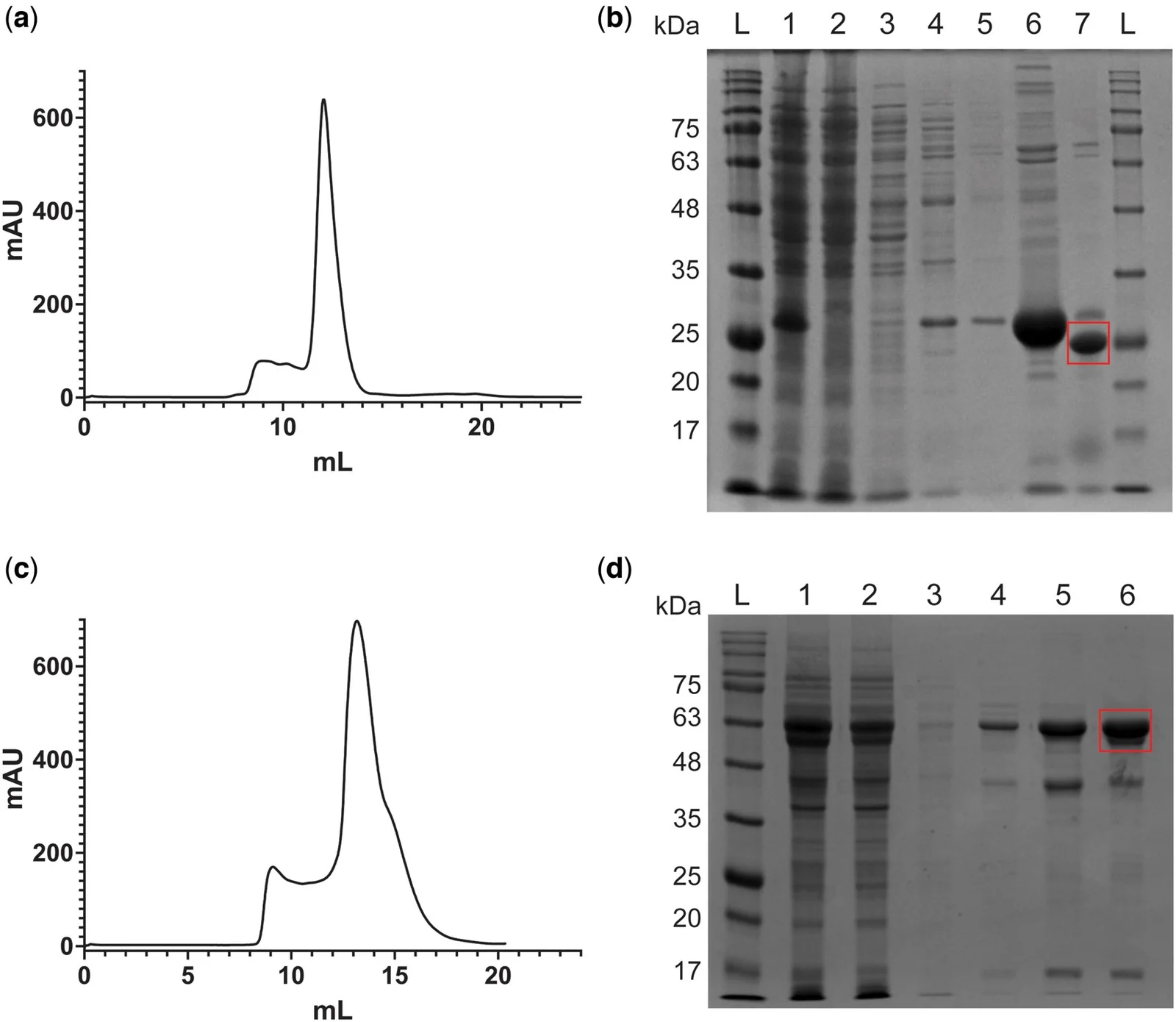
Purification of PHD2 and HIF2α ODD-GFP. (a) Following His_6_ tag cleavage, concentrated PHD2 (181–426) was applied to a Superdex75 Increase 10/300 GL column (Cytiva) generating a chromatogram plotting absorbance (mAU) against elution volume (ml). Absorbance can be used as an indicator of protein concentration. (b) Samples were taken throughout PHD2 purification and analysed via Coomassie-stained SDS-PAGE acrylamide gel. The final molecular weight of PHD2 (181–426) is 27.76 kDa and the final product is highlighted with a red rectangle. The lanes contain samples as follows: (L) BLUelf prestained protein ladder. (1) Lysate from BL21. (2) Ni-NTA agarose flowthrough. (3) Ni-NTA agarose Lysis Buffer wash. (4) Ni-NTA agarose Wash Buffer wash 1. (5) Ni-NTA agarose Wash Buffer wash 2. (6) Ni-NTA agarose elution. (7) Superdex75 pooled fractions post-thrombin cleavage. (L) BLUelf prestained protein ladder. (c) Concentrated HIF2α ODD-GFP was applied to a Superdex 200 Increase 10/300 GL column (Cytiva) generating a chromatogram plotting absorbance (mAU) against elution volume (ml). (d) Samples taken throughout HIF2α ODD-GFP purification were analysed via Coomassie-stained SDS-PAGE acrylamide gel. The final molecular weight of HIF2α ODD-GFP is blank and the final product is highlighted with a red rectangle. (L) BLUelf prestained protein ladder. (1) Lysate from BL21. (2) Ni-NTA agarose flowthrough. (3) Ni-NTA Wash Buffer wash 1. (4) Ni-NTA Wash Buffer wash 2. (5) Ni-NTA agarose elution. (6) Superdex200 pooled fractions.

1. Transform *E. coli* BL21(DE3) cells with pET-46-HIS_6_-PHD2_181 − 486_ and plate on an LB-agar plate supplemented with 100 µg/ml of ampicillin. Incubate the plate at 37°C overnight.
2. Select an isolated colony from the transformation plate and inoculate a 100 ml overnight culture in LB media supplemented with ampicillin to a final concentration of 100 µg/ml.
3. Following overnight incubation, prepare a glycerol stock from the culture and store at −80°C for future culture inoculations. (The procedure can also be started anew from Step 1 if issues with expression are observed; we observed no issues).
4. Divide the remaining overnight culture equally into 2x 1L LB cultures supplemented with ampicillin (100 µg/ml).
5. Grow the cultures at 37°C with shaking at 200 RPM until the OD_600_ reaches a range between 0.6 and 0.8.
6. Induce expression of His_6_-PHD2 with the addition of IPTG to a final concentration of 0.5 mM.
7. Grow cells for an additional 3 h postinduction at 37°C and harvest via centrifugation at 4000 RPM in a Beckman JLA-8.1000 rotor. Cell pellets can either be used immediately for purification or stored at −80°C for later use.
8. Resuspend cell pellets in Lysis Buffer supplemented with 1x SigmaFast protease inhibitor cocktail (Sigma-Aldrich).
9. Lyse cells with 3 passes through an Emulsiflex-C3 cell disruptor (Avestin) at 20 − 25 kPSI with the outlet chilled to 4°C.
10. Clarify cell lysates via centrifugation at 30 000 × g in a JA-25.50 rotor (Beckman Coulter).
11. Apply the clarified lysates to 2 ml of Ni-NTA (Thermo Scientific) resin, pre-equilibrated with Lysis Buffer.
12. Wash the Ni-NTA beads with 10 ml of Lysis Buffer.
13. Wash the beads with 30 ml of Wash Buffer 2x.
14. Elute His_6_-PHD2 from the beads by applying 10 ml Elution Buffer to the column.
15. Dialyse the 10 ml of eluted His_6_-PHD2 against 2 L of Dialysis Buffer using a 10 kDa cut-off dialysis membrane for 4 h at 4°C.
16. After 4 h, measure the concentration of His_6_-PHD2 using UV absorbance at 280 nm (ε_0.1%_ = 1.34), and add 1–1.5 units of thrombin per mg of His_6_-PHD2 to the dialysis bag.
17. Transfer the dialysis bag to 2 L of fresh Dialysis Buffer and incubate overnight at 4°C.
18. Collect the thrombin-cleaved PHD2 and apply it to 2 ml Ni-NTA resin pre-equilibrated with Dialysis Buffer to remove the uncleaved protein and cleaved His_6_ tags.
19. Collect the flow through and rinse the beads with 10 ml of Dialysis Buffer supplemented with 5 mM imidazole; Pool the flow through from this rinse with the initial flow through.
20. Concentrate the pooled sample with an Amicon 10 kDa cut-off centrifugal concentrator (Millipore) to a volume of ∼600 µl.
21. Apply the concentrated PHD2 to a Superdex 75 Increase 10/300 GL column (Cytiva) equilibrated with SEC Buffer and collect 1 column volume elution as 800 µl fractions (Fig. 3a). For purification of PHD2 for PHD2-HIF2α ODD MST experiments, add 1.2x molar equivalents of FeSO_4_ to PHD2 prior to the Superdex 200 purification.
22. PHD2 should elute as a single peak and all fractions within this peak can be pooled.
23. Analyse aliquots from each step of the purification via SDS-PAGE to assess the purity of the sample at each stage. Following elution from the Superdex 75 column, the purity of the sample should be sufficient for fluorescent labelling described below (see the ‘Fluorescent labelling of PHD2’ section).
24. If no labelling is required, aliquot and snap freeze PHD2. Store frozen PHD2 at –80°C until needed. PHD2 should not be frozen prior to fluorescent labelling.
25. Run a Coomassie-stained SDS-PAGE acrylamide gel to assess purity (Fig. 3b).

#### Fluorescent labelling of PHD2

##### Buffers

Labelling Buffer (50 mM HEPES, 100 mM NaCl, pH 7.5)

Storage Buffer (50 mM Tris-HCl, pH 7.5)

**Note:** The labelling procedure may utilize a different dye if the fluorophore is compatible with the excitation/detection wavelengths of the MST instrument at your disposal. Keep in mind that using a different dye may require re-optimizing the labelling procedure and the MST parameters to account for differences in the labelling efficiency and the intensity of fluorescent light produced by the chosen dye.

1. Dilute an aliquot of Purified PHD2 (see the ‘Purification of PHD2 catalytic domain’ section) to a concentration of 1 mg/ml with Labelling Buffer in a volume of 500 µl.
2. Resuspend a fresh 100 µg aliquot of Alexa Fluor 647 NHS ester dye (Alexa 647) in 10 µl of water and add 9 µl of the solution to the 500 µl PHD2 aliquot. This creates a dye: PHD2 molar ratio of approximately 4:1.
3. Allow the labelling reaction to proceed for 1 h in the dark at room temperature.
4. Remove excess dye by passing the sample through a PD-10 desalting column containing Sephadex G-25 resin equilibrated with Storage Buffer.
5. Remove any remaining excess dye through repeated concentration in an Amicon 10 kDa cut-off centrifugal filter followed by 10-fold dilution in Dialysis Buffer until dye is no longer detectable in the concentrator flow through by absorbance at 650 nm.
6. Measure the absorbance of the labelled PHD2 at 280 nm and 650 nm.
7. Using the below equation, calculate the corrected concentration of PHD2 accounting for conjugated dye absorbance at 280 nm. 36 900 M^−1^ cm^−1^ represents PHD2’s calculated extinction coefficient at 280 nm.
  $$[\mathrm{PHD}2]=\frac{{(A}_{280}-{0.03(A}_{650}))}{36900\;M^{-1}\;{\mathrm{cm}}^{-1}\;},$$
8. Calculate the concentration of conjugated Alexa 647 dye using the extinction coefficient of the dye at 650 nm:
  $$[\mathrm{Alexa}\;647]=\frac{A_{650}}{239,000\;M^{-1}\;{\mathrm{cm}}^{-1}\;},$$
9. Calculate the labelling efficiency of the reaction by dividing the concentration of Alexa 647 by the concentration of PHD2 to ultimately determine the number of dye molecules per protein molecule:
  $$\mathrm{Labeling}\;\mathrm{efficiency}=\frac{\left[\mathrm{Alexa}\;647\right]}{\left[\mathrm{PHD}2\right]}.$$
  This procedure should result in Alexa 647-labeled PHD2 with a labelling efficiency in the range of 1–2 dye molecules per protein molecule. Labelling efficiency in this range will provide sufficient fluorescence to detect PHD2 in the low nM range while minimally modifying the surface of the protein.
10. Bring Alexa 647-labeled PHD2 (PHD2–647) to a concentration of 10 µM in Storage Buffer.
11. Aliquot PHD2-647 and snap freeze in liquid N_2_. Store frozen PHD2-647 at −80°C until needed.

#### Purification of HIF2αODD-GFP fusion proteins

##### Buffers

Lysis Buffer (50 mM Tris-HCl, 150 mM NaCl, 5 mM Imidazole, 2 mM 2-mercaptoethanol, pH 7.5)

Wash Buffer (50 mM Tris-HCl, 150 mM NaCl, 40 mM Imidazole, 2 mM 2-mercaptoethanol, pH 7.5)

Ni-NTA Elution Buffer (50 mM Tris-HCl, 150 mM NaCl, 500 mM Imidazole, 2 mM 2-mercaptoethanol, pH 7.5)

SEC Buffer (50 mM Tris-HCl, 100 NaCl, pH 7.5)

1. Transform *E. coli* BL21(DE3) cells with pET21b-HIF2α-ODD-GFP-35-His_6_ and plate on an LB-agar plate supplemented with 100 µg/ml of ampicillin. Incubate the plate at 37°C overnight.
2. Follow steps 2 − 7 from the ‘Purification of PHD2 catalytic domain’ section to generate pET21b-HIF2α-ODD-GFP-35-His_6_ cell pellets.
3. Resuspend cell pellets in Lysis Buffer supplemented with 1X SigmaFast protease inhibitor cocktail (Sigma-Aldrich).
4. Lyse cells with three passes through an Emulsiflex-C3 cell disruptor (Avestin) at 20 − 30 kPSI with the outlet cooled to 4°C.
5. Clarify lysates via centrifugation at 30 000 × g for 45 minutes in a JA-25.50 rotor (Beckman Coulter).
6. Apply the clarified lysate to a column containing 2 ml Ni-NTA beads pre-equilibrated with Lysis Buffer.
7. Wash the beads twice with 30 ml Wash Buffer.
8. Elute HIF2αODD-GFP-His_6_ from the beads with 6 ml of Elution Buffer.
9. Concentrate the eluted protein using an Amicon centrifugal filter with a 30 kDa cut-off membrane to a volume of ∼600 µl.
10. Apply the sample to a Superdex 200 Increase 10/300 GL column (Cytiva) pre-equilibrated with SEC buffer.
11. HIF2αODD-GFP-His_6_ will elute as a single peak, note that the elution volume will be lower than expected of a globular protein with the same molecular weight but will be within the separation range of the Superdex 200 column (there shouldn’t be much material eluted with the void volume).This is likely due to an extended conformation of the HIF2αODD caused by an expected lack of secondary structure in this sequence (Fig. 3c).
12. Pool fractions corresponding to HIF2αODD-GFP-His_6_.
13. Adjust the protein concentration of the sample with SEC Buffer to a final concentration of 10 µM.
14. Aliquot protein samples and snap freeze in liquid nitrogen.
15. Store frozen HIF2αODD-GFP-His_6_ at −80°C until needed.
16. Run a Coomassie-stained SDS-PAGE acrylamide gel to assess purity (Fig. 3d).

### MST experiments

#### PHD2-HIFα peptide

##### Materials and buffers

PHD2-647

HIF2α CODD Peptide (Ordered from GenScript)

PHD2 Dilution Buffer (50 mM Tris-HCl, 5 mg/ml BSA, pH 7.5)

Peptide Dilution Buffer (50 mM Tris-HCl pH 8.0)

10 mM FeSO_4_ Stock solution (Dissolved immediately before addition to PHD2-647) (100x Stock)

100 mM Stock Solution of N-Oxalylglycine (NOG) (100x Stock)

**Figure 4:**
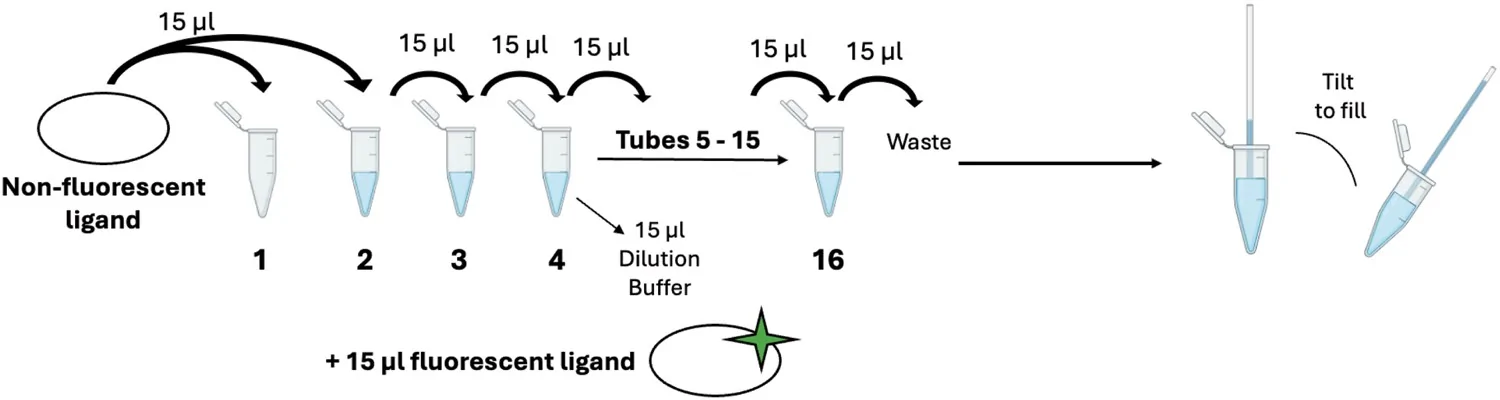
Experimental set up of an MST replicate. To create a titration series of the nonfluorescent ligand (WT HIF2α CODD peptide or PHD2), a two-fold serial dilution was performed. Fifteen microliters of nonfluorescent ligand was added to tube 1 and 15 µl of buffer was added to tubes 2–16. Fifteen microliters of nonfluorescent ligand was added to tube 2 and mixed thoroughly. Fifteen microliters of solution was removed from tube 2 and transferred to tube 3 followed by thorough mixing. This process was repeated through tube 16, where 15 µl of solution was removed and discarded so that each tube contained 15 µl total. Fifteen microliters of fluorescent ligand (PHD2–647 or HIF2α ODD-GFP) was added to each tube. A capillary was placed into each tube, tilting as shown to completely fill.

1. Turn on the MST instrument to allow time for initialization and stabilization while the materials for the experiment are prepared.
2. Thaw an aliquot of 10 mM PHD2-647 by hand.
3. Once completely thawed, centrifuge the aliquot in a desktop microfuge at 4°C for 10 minutes to ensure the removal of any aggregates caused by freeze thawing. The presence of very low amounts of aggregated material in a sample can cause issues with MST data acquisition and thawed aliquots should always be centrifuged prior to use.
4. Dilute PHD2-647 to a concentration of 50 nM in PHD2 Dilution Buffer and store on ice. 1 ml of solution is an appropriate amount to prepare for 3 replicates of affinity measurements.
5. Add FeSO_4_ to the diluted PHD2-647 solution to a final concentration of 10 µM and add NOG to a final concentration of 1 mM.
6. Dissolve ∼1 mg HIF2α CODD peptide in Peptide Dilution Buffer and store on ice.
7. Measure the concentration of the peptide by absorbance at 280 nm, utilizing the calculated extinction coefficient of the peptide. If no W or Y residues were present in the peptide sequence, a BCA (Thermo Scientific) assay can be used to determine the concentration of the peptide. Alternatively, the concentration can be determined by measuring absorbance at 205 nm, with careful notice to buffer interference at this wavelength [29].
8. Set up 16x 250 µl PCR tubes or tube strips in a tube rack.
9. Leave the first tube empty and add 15 µl of Peptide Dilution Buffer to the 15 remaining tubes.
10. Add 15 µl of peptide to the first tube; this tube is empty.
11. Create a 2-fold serial dilution of the peptide by adding 15 µl of peptide to the second tube (containing 15 µl of buffer) and mix thoroughly by pipetting up and down. Remove 15 µl of solution from the second tube and transfer to the third tube; mix thoroughly via pipetting. Repeat this process across the remaining tubes, discarding 15 µl from the last tube so the final volume in every tube is 15 µl (Fig. 4a).
12. Add 15 µl of 50 nM PHD2-647 to each of the 16 tubes and mix thoroughly by pipetting. Incubate the tubes for at least 15 minutes at room temperature.
13. Place 1 Standard MST Capillary into each tube. The tubes will fill via capillary action. Check each capillary to ensure they are completely full before loading onto the tray. If the capillary is partially filled, tilt and gently tip them, so the capillary and tube are slanted to allow the solution to more easily flow to the other end (Fig. 4).
14. Place each filled capillary onto the MST sample tray starting from the highest peptide concentration to the lowest.
15. Load the MST sample tray into the instrument.
16. In the M.O. Control software, select the appropriate excitation light for the dye (Red for Alexa 647).
17. Set the Excitation power to 20% and the IR laser power to medium.
18. Perform a capillary scan to check for sample issues.
19. Adjust the excitation power if there is too much (>1000 counts) or too little (<200 counts) fluorescence. Repeat the scan if the fluorescence power is altered. If the variation in the fluorescence counts between the capillaries is >20% from the average, halt the experiment, and redo the serial dilution. Variations in fluorescence counts are likely due to pipetting error. MO. control displays this information.
20. Set up an affinity measurement by inputting the concentrations of PHD2-647 and the peptide solutions. Set the MST time trace parameters as:
  - Pre-IR time: 5 seconds
  - IR on time: 20 seconds
  - Post-IR time: 3 seconds
    - A longer post-IR time (e.g. 30 seconds) may be recommended to serve as a quality-control step in the case of irreversible unfolding and/or aggregation of the protein due to heating by the IR laser [30]. If the post-IR steady state is vastly different from the baseline steady-state, protein structure may have been altered, thus compromising the interpretation of the binding affinity.
21. Record MST traces.
22. Discard used capillaries in a sharp waste container. Perform 2 replicate experiments using the above protocol. Data analysis will be discussed below.

#### PHD2-HIFαODD

##### Materials and buffers

PHD2 (100 µl of ∼1 mM PHD2 required for three replicates)

10 µM HIF2α-ODD-GFP-His_6_ aliquot

PHD2 Dilution Buffer (50 mM HEPES, 150 mM NaCl, pH 7.5)

HIF2α Dilution Buffer (50 mM Tris-HCl, 150 mM NaCl, 2 mM 2-mercaptoethanol, 0.1% Tween 20 pH 7.5)

10 mM FeSO_4_ Stock solution (Dissolved immediately before addition to PHD2-647) (100x Stock)

100 mM Stock Solution of N-Oxalylglycine (NOG) (100x Stock)

1. Turn on the MST instrument to allow time for initialization and stabilization while the materials for the experiment are prepared.
2. Thaw an aliquot of PHD2 and 10 µM HIF2α by hand.
3. Once completely thawed, centrifuge the aliquots in a desktop microfuge at 4°C for 10 minutes to ensure the removal of any aggregates caused by freezing and thawing.
4. Dilute HIF2α-ODD-GFP-His_6_ to a concentration of 40 nM in HIF2α Dilution Buffer and store on ice. One millilitre of solution is an appropriate amount to conduct three replicates of affinity measurements.
5. If not already done, determine the concentration of PHD2 using the absorbance at 280 nm (extinction coefficient of 36 900 M^−1^ cm ^−1^) and adjust the concentration to ∼1 mM.
6. Supplement the PHD2 solution with NOG to a final concentration of 2 mM and incubate on ice for 10 min.
7. Remeasure the concentration post-adjustment and NOG addition for input in MO. control software.
8. Set up 16x 250 µl PCR tubes or tube strips in a tube rack.
9. Leave the first tube empty and add 15 µL of PHD2 Dilution Buffer to the 15 remaining tubes.
10. Add 15 µL of PHD2 to the first tube; this tube is empty.
11. Create a 2-fold serial dilution of PHD2 following the same procedure as described in the ‘PHD2-HIFα peptide’ section, step 11 (Fig. 4).
12. Add 15 µL of 40 nM HIF2α-ODD-GFP to each of the 16 tubes and mix thoroughly by pipetting up and down. Incubate the tubes for at least 15 minutes at room temperature.
13. Prepare and load MST capillaries as described in the ‘PHD2-HIFα peptide’ section, steps 13–15.
14. In the M.O. Control software, select the appropriate excitation light for the dye (Blue for GFP).
15. Set the Excitation power to 20% and the IR laser power to medium.
16. Perform a capillary scan and check the fluorescence counts as described in the ‘PHD2-HIFα peptide’ section, steps 18–19.
17. Set up an affinity measurement by inputting the concentrations of HIF2α-GFP-ODD and PHD2 solutions. Set the MST time trace parameters as:
  - Pre-IR time: 5 seconds
  - IR on time: 20 seconds
  - Post-IR time: 3 seconds
18. Record MST traces.
19. Discard used capillaries in a sharp waste container. Perform 2 replicate experiments according to the above protocol. Data analysis will be discussed below.

### Data analysis

1. The data files recorded by NanoTemper’s MST instruments can be directly read and analysed by their own software suite M.O Affinity analysis or the analysis tool PALMIST.
2. Load the data file into your chosen software suite and average replicate *F*_n_ measurements and determine measurement errors.
3. Fit the 1:1 *K_d_* model to the averaged data and determine the *K_d_* value.

### M.O. affinity analysis (NanoTemper)

M.O. affinity analysis is NanoTemper’s proprietary software provided with the instrument. It can accept the data files output by the instrument directly for analysis making using the software convenient if only straightforward analysis is required. It allows for the fitting of the 1:1 *K_d_* model to data as well as the Hill model (not discussed here). Additionally, the software allows for the review of quality check data recorded within the NanoTemper data files such as capillary scans, initial fluorescence and bleaching rates.

To analyse PHD-HIF interactions a data file(s) containing three replicate curves can be loaded and added into the “data selection” pane as a “merge set” which will automatically average the data points and determine experimental error. In the “dose response fit” pane, not much needs to be altered aside from the default time post IR laser “on time” used for the measurement’s fluorescence measurement point. The software uses 15 seconds after IR laser activation as a default value which may or may not be appropriate for every sample. We would recommend changing the time point used to line up with the flat part of the curve (steady-state) for your samples. For HIF2α CODD+PHD2, 20 seconds on time was selected. Ensure that the *K_d_* model is selected and the target concentration (TargetConc variable) is set to be fixed to the known concentration of fluorescent protein. Results can be viewed and exported from the “compare results” pane. Multiple data sets can be loaded and analysed at once and exported together. Following export from M.O. software, we input data into Prism10 and fit the data with the “One site-Specific binding” nonlinear regression equation to generate a figure.

### PALMIST

Another MST analysis program is PALMIST (Python-based AnaLyzer of MIcroScale Thermophoresis) developed by Brautigam’s group [20]. This program is free to download and is distributed with an easily accessible manual. PALMIST loads time trace files from M.O. Control (.ntp files), automatically performs necessary calculations, and generates an isotherm graph ready for further analysis. Advanced analytical features not available on M.O. affinity analysis are offered on PALMIST such as in-depth statistical analysis and a unique two-site binding model. While our analyses were performed in M.O. affinity analysis, future work may benefit from a deeper analysis in PALMIST. Papers published by the group responsible provide more information on this program if desired [20, 31].

## Results and discussion

In our previous work, we were able to assess the impact of 18 mutations in the HIF2α CODD region on the interaction between PHD2 and HIF2α [16]. This dataset defined some useful boundaries which may guide the assessment of pathogenicity. Using the protocol described above (see the ‘PHD2-HIFα peptide’ section), it is possible to evaluate novel Pacak–Zhuang HIF2α mutations as they are reported and compare them with previously analysed and classified mutations. Most HIF2α mutations seem to cluster around the CODD site [15], hence the use of a HIF2α CODD peptide. Despite this, there is rationale for a binding assay with a more intact HIF2α construct. In addition to the need to evaluate mutations outside of the CODD region, analysing HIF2α-PHD2 binding in a full ODD context may reveal more physiological details about the interaction than a peptide study can provide.

**Figure 5:**
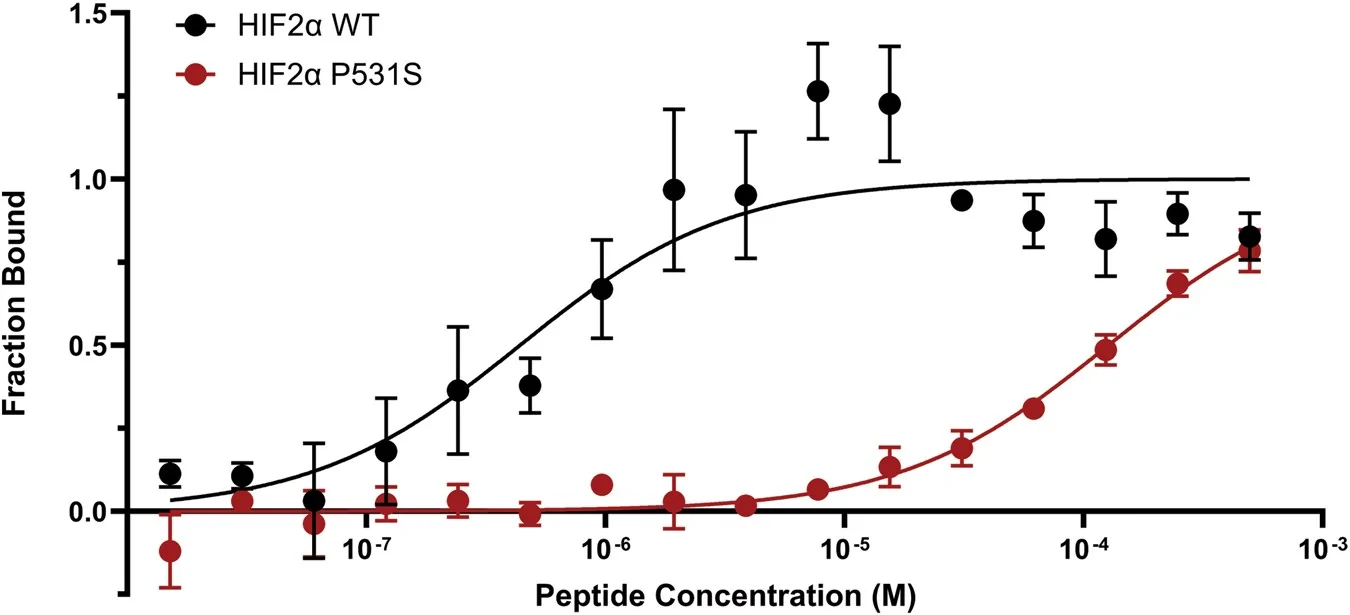
Class 1 Pacak–Zhuang syndrome HIF2α mutation impairs binding to PHD2 in a full ODD context. Binding between PHD2 and HIF2α ODD-GFP was measured using MST. Binding curves of HIF2α ODD WT (black) and HIF2α ODD P531S (red) with PHD2 were generated. As expected, P531S, a clinically reported Class 1 Pacak–Zhuang mutation, has a severe binding defect with PHD2 in a full ODD context. Experimental data points are indicated by solid circles and the curves of the *K_d_* functions fit to the data points are represented by solid lines. Error bars show the standard deviation of experiments performed in triplicate.

Following the above MST protocol (see the ‘PHD2-HIFαODD’ section), we tested the feasibility of a HIF2α ODD—PHD2 binding assay by comparing the binding affinity of PHD2 for HIF2α ODD WT to a well reported Class 1 Pacak–Zhuang mutation, HIF2α ODD P531S^4^ [12, 26, 32–35]. We observed nearly a 300-fold decrease in the binding affinity of PHD2 for HIF2α ODD P531S in comparison to WT with *K_d_* values of 130 ± 30 μM and 400 ± 200 nM, respectively (Fig. 5). These measurements follow the trends of the *K_d_* values determined in the context of the HIF2α CODD peptides, in which we determined a *K_d_* of 34 ± 2 μM for HIF2α CODD WT and no binding for HIF2α CODD P531S [1]. The *K_d_* values of the HIF2α-ODD constructs were markedly lower than those of the HIF2α CODD peptides, indicating a higher binding affinity for PHD2 in a full ODD context. The HIF2α ODD construct likely makes additional contact with PHD2 in a manner that is not observed with the shorter HIF2α CODD peptide. This may indicate a HIF2α ODD-PHD2 binding assay with a longer ODD construct is more physiologically relevant than a peptide binding assay. However, HIF2α peptides are beneficial for their utility and easy procurement.

Since HIF2α binding defects for PHD2 are correlative with Pacak–Zhuang disease severity, it may be possible to predict disease outcome simply by measuring novel HIF2α mutant *K_d_* values for PHD2 and compare them against our previously reported HIF2α mutant binding values [16]. To help achieve that goal, we present an effective and accessible method for measuring binding between PHD2 and HIF2α. MST is an untethered binding technique with modest sample requirements, making it an ideal experimental approach for evaluating HIF2α mutations as they are reported. We have optimized two methods to measure HIF2α-PHD2 binding using MST, one using a HIF2α CODD peptide and the other with HIF2α ODD protein construct. These methods present a promising protocol for establishing disease outcome of novel and existing Pacak–Zhuang Syndrome mutations.

## Data Availability

The authors declare that the data represented in this article are included in the article. Further data are available from the corresponding author upon reasonable request.
